# Quack Advertisements

**Published:** 1848-07

**Authors:** 


From the American Journal and Library of Dental Science.

### QUACK ADVERTISEMENTS.

It is an unpleasant, and generally a profitless task to either to speak, write, or in any other manner lift the warning voice against that spe ies of nuisance known as quack advertisements. We say, unpleasant is the duty of him who undertakes it, for if he discharges it properly, his conscience, and every principle of justice, honesty, and humanity, require that he should use the strongest, boldest and most uncompromising language of censure, and profitless, for the people in whose behalf he undertakes it, gives so little heed to the warning admonition.

But this forms no excusable ground for shrinking from the duty. Quack advertisements—to say nothing of the quacks themselves—we assert are the means of destroying daily an incalculable number of the lives, health and happiness of the people—that they have not a single element of good in them—but are only an evil, and that continually.

To guard therefore against imposture, we will give, what we hold to be, the cardinal elements entering into their composition, and by which their true character may always be recognised and detected.

The *first most prominent element* is ITS CERTAIN INFALLIBILITY in every case, to the accomplishment of the object it proposes. Does it relate to the diseases of the human frame? The self-styled advertising physician asserts, without qualification, an infallible cure for the whole. Or if to a particular part of the body, as for example, the teeth, an infallible, would-be-dentist gives most positive and unequivocal assurance that he can make, adjust, and replace teeth in a manner that can hardly be equalled by *nature herself*, but further, attempts even to put her to the blush.

*The second element* characterising quack advertisements, is, namely, their *unblushing impudence, falsehood and villainy*, for at a single sweep, they set aside, and put at defiance all the accumulated knowledge of the learned, wise and good of the present and past ages, and falsely, unblushingly, and wickedly assert that the whole is nothing, and worse than nothing, in comparison with their own instinctive or improved, infallible and never failing remedies.

*The third element* characterising quack advertisements is the universal ignorance or knavery of their authors.

Has any one been deceived? Let him inquire after the qualifications of his deceiver, and he will find him an illiterate, ignorant pretender, or otherwise an accomplished and designing knave.

These three elements constitute, we think, the great distinguishing marks of quack advertisements. We would therefore call upon the people as they value the beauty and soundness of their teeth—as they value their health, their happiness, and their lives, to seriously reflect upon the remarks we have just made, and we not only respectfully ask, but affectionately challenge, their honest reflection—and if they be true, then to shun all such advertisements as they would a viper, as they would their worst enemy. Quackry is always busy. It is never idle, neither by night nor by day; it is continually planning means by which to deceive the credulous and unsuspecting. Almost every newspaper in the country contains a glowing account of some wonderful and marvellous cure effected by some one of its thousand nostrums.

